# Lipid Extracts Obtained by Supercritical Fluid Extraction and Their Application in Meat Products

**DOI:** 10.3390/antiox11040716

**Published:** 2022-04-05

**Authors:** Branislav Šojić, Predrag Putnik, Bojana Danilović, Nemanja Teslić, Danijela Bursać Kovačević, Branimir Pavlić

**Affiliations:** 1 Faculty of Technology, University of Novi Sad, 21000 Novi Sad, Serbia; sojic@tf.uns.ac.rs; 2 Department of Food Technology, University North, 48000 Koprivnica, Croatia; pputnik@alumni.uconn.edu; 3 Faculty of Technology, University of Niš, 16000 Leskovac, Serbia; bojana.danilovic@junis.ni.ac.rs; 4 Institute of Food Technology, University of Novi Sad, 21000 Novi Sad, Serbia; nemanja.teslic@fins.uns.ac.rs; 5 Faculty of Food Technology and Biotechnology, University of Zagreb, 10000 Zagreb, Croatia

**Keywords:** meat products, supercritical fluid extraction, lipid extracts, natural antioxidants, antimicrobials

## Abstract

Supercritical fluid extraction (SFE) has been recognized as the green and clean technique without any negative impact on the environment. Although this technique has shown high selectivity towards lipophilic bioactive compounds, very few case studies on the application of these extracts in final products and different food matrices were observed. Considering the recent developments in food science and the increasing application of supercritical extracts in meat products in the last decade (2012–2022), the aim of this manuscript was to provide a systematic review of the lipid extracts and bioactives successfully obtained by supercritical fluid extraction and their application in meat products as antioxidant and/or antimicrobial agents. Lipophilic bioactives from natural resources were explained in the first step, which was followed by the fundamentals of supercritical fluid extraction and application on recovery of these bioactives. Finally, the application of natural extracts and bioactives obtained by this technique as functional additives in meat and meat products were thoroughly discussed in order to review the state-of-the-art techniques and set the challenges for further studies.

## 1. Introduction

In 2020, modern meat market was USD 838 billion, and it is expected to further increase up to USD 1 trillion by 2025. During the same year, 328 million tons of meat were produced in the world, which is equal to 35 kg of meat/capita. Hence, meat market has large share in international food industry [1]. One of the largest challenges in meat processing is oxidation processes that are dangerous for human health and the market value of meat products.

Oxidative reactions (lipid and protein oxidation) and the growth and activity of microbial populations are the two main causes of deterioration in the quality of meat and meat products [2]. Due to the relatively high content of unsaturated lipids and the presence of a variety of oxidants in muscles, meat and meat products are highly susceptible to lipid and protein oxidation. Hydroperoxides as primary and volatile compounds (e.g., aldehydes, ketones, organic acids) as secondary products of lipid oxidation led to a reduction in nutritional content and shelf-life, as well as market value of the final products [3,4,5]. Moreover, oxidative reactions in muscle protein have a strong influence on the overall quality of meat and meat products. Muscle peptides in interacting with reactive oxygen species lead to the formation of covalent intermolecular cross-linked proteins, resulting in protein aggregation and fragmentation. The changes in proteins in muscles promote the reduction in water-holding capacity, loss of biological functionality of proteins, and reduction in sensory quality (colour, flavour and texture) of meat and meat products [6,7,8,9]. Generally, the prevention of oxidative reactions (e.g., lipid and protein oxidation) in the meat products is a key challenge for meat processing technology. Lipid oxidation in meat and meat products is usually evaluate by measuring the amount of peroxide value (PV), thiobarbituric acid-reactive substances (TBARS), whereas the sulphydryl and carbonyl group generated during the meat processing and storage show the level of protein oxidation [8]. 

Depending on the initial hygiene and the preservation method used, the growth of microbial population (spoilage and pathogenic) can cause spoilage and accordingly various foodborne poisonings in meat and meat products [9]. The growth of spoilage bacteria, yeasts and molds can decompose the main components of meat (e.g., lipids and proteins, and vitamins) and led to slime, unpleasant flavour and abnormal discolouration that reduced the shelf-life of the final products [2]. 

The pathogenic bacteria (e.g., *Salmonella* spp., *Escherichia coli* O157:H7, *Listeria monocytogenes*) are primarily responsible for numerous foodborne diseases and food poisoning, which is why they are considered the most important limiting factor in the meat industry [9].

To prevent negative changes (chemical and microbial) during processing, meat processors usually use numerous additives (e.g., antioxidants and antimicrobials) [1]. However, most of these additives are synthetic and may have adverse effects on human health. For example, some meat products are classified as Group 1 carcinogens by the World Health Organisation (WHO) due to the use of nitrites and nitrates, which are very common in meat processing [8,10]. They are added to meat as an important antioxidant that prevents the growth of bacteria and off-flavours while preserving the appealing colour of the products. Namely, the use of nitrites and nitrates contributes to the of development of the typical reddish-pink colour and flavour of cured meat products. On the other hand, nitrites and nitrates promote the formation of N-nitrosamines, which are associated with cancer risks. The current Serbian legislation has restricted the maximum amount of nitrate or nitrite that may be added in processed meat expressed as NaNO_2_ or NaNO_3_ to 100 and 150 mg/kg, depending on the type of product. Besides nitrites and nitrates, there are other synthetic additives such as butylated hydroxytoluene, butylated hydroxyanisole, tertiary butyl hydroquinone and propyl gallate, which are used to ensure microbial safety and prevent oxidation of products, but also have carcinogenic effects on humans [11,12]. Therefore, in the last decade, the use of natural antioxidants and antimicrobial agents, isolated from various plant materials has received considerable attention in meat processing [2,13]. The natural compounds used as antioxidants and antimicrobial agents in meat products are mainly various essential oils (EOs) or plant extracts [2,4,8]. The ability of these compounds to provide oxidative stability and protection against pathogenic and spoilage microorganisms in meat products has been extensively studied. 

Most research has focused on the use of medicinal and aromatic plants with proven antioxidant and antimicrobial potential [14,15,16], but in recent years there has been an increasing use of fruit extracts [17,18,19]. In addition to conventional extraction methods, supercritical fluid extraction (SFE) has proven to be a useful method to obtain extracts of high quality and good biological activity, rich in phenolic and flavonoid compounds. Moreover, supercritical fluid extracts do not contain solvent residues and preserve the aroma of the plant [13].

Nutraceuticals and bioactive compounds can be isolated from natural resources by various extraction techniques before further use as natural additives in food matrices. Conventional extraction processes (Soxhlet extraction, hydrodistillation, etc.) can be effectively used for the recovery of lipophilic bioactive compounds. However, the use of these methods is decreasing, which is related to their limitations. The major problems with traditional extraction techniques are the use of expensive and toxic organic solvents (hexane, methylene chloride, etc.), poor yield and quality of the final product, and huge consumption of time, energy and resources.

The recent trend and challenges posed by green chemistry place high demands on chemical engineering technologies. The most important aspects have been defined by Chemat et al. [20] and are related to the use of alternative solvents that are non-toxic, non-flammable and without toxic residues in the obtained herbal extracts, the use of renewable resources or plant cultivation instead of uncontrolled harvesting of natural resources and the discovery and development of new extraction processes with non-hazardous solvents, low energy requirements, low cost, renewable natural products and high-quality extracts with bioactive molecules. Classical extraction methods are overwhelmed with the above requirements, whereas emerging extraction techniques are increasingly capable of meeting these requirements. Within the concept of green extraction, novel technologies are being developed that include various extraction techniques such as high-pressure extraction (supercritical or subcritical fluid extraction), microwave-assisted extraction, ultrasound-assisted extraction, extraction accelerated by pulsed electric fields, enzyme-assisted processes and extraction with natural deep eutectic solvents.

SFE has been particularly recognized as the green and clean technique with no negative impact on the environment. Moreover, this technique has shown high selectivity towards lipophilic bioactive compounds and the ability to obtain extracts with concentrated target compounds without traces of potentially harmful solvents. De Melo et al. [21] provided an extensive literature review on the application of SFE to isolate a wide range of bioactive compounds from natural sources, whereas Essien et al. [22] reviewed in detail the recent advances in the field of SFE application. An enormous number of studies have been carried out by the scientific community focusing on the SFE of different plant matrices with the aim of determining the extraction kinetics and understanding the phenomena occurring in this process, maximizing the yield of the target compound(s), determining the chemical profile and/or evaluating the bioactivity of the extracts, and finally optimizing the process and adapting it for industrial production. However, there are very few case studies on the application of these extracts in final products and different food matrices. A good example is the case study on sage (*Salvia officinalis* L.) herbal dust, where the SFE study started with the evaluation of the process kinetics [23], determination of chemical profile and in vitro bioactivity [24], which was followed by the application of these extracts obtained by SFE as natural additives in fresh pork sausages [25], minced pork meat [26] and fresh kombucha cheese [27].

Considering the recent developments in food science and the increasing application of supercritical extracts, the aim of this manuscript was to provide a systematic review of the lipid extracts and bioactives successfully obtained by supercritical fluid extraction and their application in meat products as antioxidant and/or antimicrobial agents.

## 2. Methodology

This literature review focused on commonly used sources of scientific references as Web of Science, PubMed, Scopus, and ScienceDirect, together with usual internet search engines as Google.com, Bing.com, Yandex.com and Baidu.com. Time period was approximately set to 2012–2022 (accessed on 03.11.2021); however, more concern than time restraints was given to adequate coverage of the topics from the manuscript as types and origin of lyophilic bioactives, principles and parameters of SFE, and application of bioactives in meat products. 

## 3. Lipophilic Bioactives from Natural Resources 

The use of plant essential oils (EOs) has a long tradition in humankind, and they are usually obtained as secondary metabolites from various families of plants including *Asteraceae, Lamiaceae, Lauraceae, Myrtaceae, Rutaceae* and others (e.g., citruses) [13,28,29]. The most common origins of Eos include rosemary, oregano, sage, thyme and basil [28,29]. They are volatile oily substances that vary in colour and their properties are dependent of their chemotype, pedoclimatic conditions, harvesting, processing etc. [30]. Conducted literature reviews of scientific reports revealed that chemical profiling of the plant products for application in meat processing usually involves the extraction of Eos and SFE extracts. Hence, that will be used for further commenting of results.

The Eos and extracts are primarily composed of various groups of important compounds for meat industry due to their antimicrobial and antioxidative abilities, mainly referred to terpenoids (e.g., camphor, camphene, carvacol, thymol, etc.), phenylpropanoids (caffeic, ρ-coumaric, ferulic, rosmarinic and sinapic acids), carotenoids (lycopene, β-carotene, phytofluene, phytoene and lutein), tocochromanols (tocopherols and tocotrienols), and organosulfur compounds (allicin, alliin, etc.). Terpenoids are highly variable group of compounds also known as isoprenes, which are widely represented in plants while being formed from five carbon isoprene units. Polyphenols are secondary plant metabolites with minimum of one aromatic ring that has attached one (or more) hydroxyls groups [31]. Carotenoids are composed of 3–13 conjugated double bonds, also called polyenes with six-carbon ring structures [32]. Tocochromanols or chroman-6-ols includes a group of molecules with chromane ring and tocochromanols [33]. Organosulfur compounds are potent group of organic molecules that are mostly lipophilic and contain sulfur.

As mentioned above, one of the main qualities for assessment of lipophilic bioactives for usage in meat industry pertains to their antimicrobial and antioxidant potentials [34]. However, exact mechanisms of terpenoid antimicrobial activity are currently debated [30], but still for all lipophilic molecules it was proposed that they target cellular membrane’s structures causing damages to microbes. For example, they may target ion transport channels, interfere with respiration or structural permeability and affect drops of pH [30]. Additionally, their fat-soluble character allows them to be more readily incorporated into meat matrices which is one of the advantages for meat industry [30]. 

Aside from terpenoids (that also have antioxidative roles), polyphenolic compounds are another important group of major carriers of antioxidative activity in plant material. Main groups of polyphenols with desired qualities for meat industry include flavonoids, phenolic acids, stilbenes, coumarins, lignans, quinones, and curcuminoids [35]. For instance, it was found that the SFE extracts from wild thyme (*Thymus serpyllum* L.) applied to pork patties mainly contained terpenoids carvacrol (40%) and thymol (15–16%) with lesser quantities of α-terpineol, borneol and geraniol [36]. Furthermore, Winter savory (*Satureja montana* L.) Eos that were used to extend the storage of fresh pork sausages mainly contained carvacrol (59%) with smaller amounts of thymol and polyphenolic 3,5-dimethylphenol. SFE extracts from the same plant also contained carvacrol (70.92% and 46.32%) where its content was strongly increased with higher pressure and temperature [37]. Furthermore, oregano (*Origanum vulgare*) SFE extract contained tetrahydroxy-dimethoxyflavone derivative, rosmarinic acid, dihydroxy-trimethoxyflavone derivative, sakuranetin, velutin, luteolin-methyl ether, xanthomicrol, genkwanin, and diosmetin 7-*O*-rutinoside [38]. It was also proposed that tamarillo extract (*Solanum betaceum* Sendtn), due to its high content of polar polyphenols, is able to prevent lipid oxidation in cooked beef meat [39]. 

Additional lipophilic polyphenol that originates from peanuts (*Rachis hypogea*) and grapevines (*Vitis vinifera*), and Japanese knotweeds (*Polygonum cuspidatum)* is resveratrol. It is also present in red wine and mulberries (*Morus*). This chemical is also abundantly existent in grape pomace that is byproduct from winemaking industries (and potential raw material for its production), that possibly can increase meat quality (e.g., oxidative stability of minced camel meat) [40]. Similarly, this lipophilic polyphenol can serve as potential antioxidants in cooked foods for providing better oxidative stability of ground pork [41].

The next group of important lipophilic compounds originating from plant material are carotenoids (largely used as colourants and antioxidants) [42,43]. One of well-known carotenoid that holds great significance for meat industry is bixin (colorant for sausages and meats), commonly found in Annatto seeds (*Bixa Orellana* L.) [44]. Bixin and norbixin are recognized as additives in European Union and labeled as E160b [44]. It was found that with manipulating temperature and pressure in SFE it was possible to recover up to 45% of these carotenoids in the extracts. Other important carotenoids include lycopene, β-carotene, phytofluene, phytoene and lutein [43]. They are best extracted from plants that are their rich sources as tomatoes (*Solanum lycopersicum*) or carrots (*Daucus carota*) and their byproducts. As expected, extracts with higher contents of carotenes will have lipophilic nature. With SFE of tomato byproduct it is possible to produce leoresin with high content of resin and essential oil that is rich in carotenoids and useful for the production of beef hamburgers [43].

Tocochromanols are important antioxidants and vitamins that are almost equally interesting for human health and food production. Vitamin E is one of the lipophilic tocos that is a fat-soluble molecule with eight different structures, where four of them are tocopherols and another four are tocotrienols [43]. Raw materials for vitamin E can be green plants and the vegetables oil from where it can be extracted with SFE [43].

The Eos made from garlic (*Allium sativum*) and other *allium* members are rich with lipophilic organosulphur compounds with antioxidant and antimicrobial qualities [34]. The most important representative is allicin that is commonly associated with various antimicrobial potentials that can be additive for various products, as minced meat [45,46,47], Brazilian low-sodium frankfurters, and pastrami.

After the brief description of the most important lipophilic bioactives and their natural sources for meat industry, in the next lines it will be discussed their extraction with SFE technology.

## 4. Supercritical Fluid Extraction of Lipophilic Bioactives

### 4.1. Basic Principles of SFE

Solid–liquid extraction (SLE) using organic solvents such as toluene, hexane, heptane, petroleum ether, dichloromethane, chloroform, ethanol, methanol, etc., is a traditionally accepted procedure for the recovery of various classes of lipophilic compounds. This process is usually carried out at lower temperatures than conventional hydrodistillation (HD), thus reducing the risk of possible chemical changes and/or degradation of target compounds due to elevated temperatures. Although non-polar organic solvents have good affinity and selectivity for lipophilic bioactives (essential oils, carotenoids, non-polar polyohenols), concomitant lipophilic compounds such as waxes may be co-extracted, reducing the content of target compounds in the extracts. The use of organic solvents in SLE is associated with other major problems such as particularly high costs due to the consumption of expensive resources, poor environmental impact due to the discharge of the chemicals used, and the major risk of human toxicity that may occur during production or due to the traces of the solvents in extracts and final products.

On the other hand, HD is the most commonly used method for isolating non-water-soluble compounds with high-boiling points such as Eos, which consists only of volatile compounds. This technique is the most widely used in the world due to its simplicity, it is considered the most cost-effective technique for the production of most Eos at a reasonable price in less developed countries [48]. Although HD is already an established and widely used procedure for isolating pure Eos, this technique has some disadvantages in terms of chemical changes such as hydrolysis and thermal degradation that occur during the process and can affect the quality and bioactivity of the product. Moreover, this process consumes a lot of energy for steam generation, heating and cooling. Since the process can take several hours, it is very time-consuming, and prolonged contact between the fresh or dry plant matrix and the heated water increases the risk of undesirable chemical changes and thermal degradation of Eos [48].

SFE has been developed as an environmentally friendly and clean technique that can overcome the above challenges associated with SLE and is an excellent alternative and a current method of choice for the isolation of lipophilic compounds. Over the last 40 years, this technique has become increasingly important in the food, pharmaceutical and cosmetics industries. The most important aspects of these improvements over SLE and HD are reduced solvent consumption, preservation and reuse of energy, reduced time, improved selectivity and total yield, and avoidance of degradation of bioactive compounds by high temperatures.

The supercritical state of matter is associated with a tremendous effect on its physico-chemical properties, which is the main principle of SFE. For example, the increase in pressure leads to an increase in the density of supercritical fluid, whereas some of its transport properties (diffusivity, surface tension and viscosity) are more similar to those gasses [22]. The low viscosity and enormous diffusivity of the supercritical fluid led to an improvement in the heat and mass transfer coefficients of pressurized fluids and consequently to better penetration into the pores of the plant material and rapid dissolution of the target compounds. Among the liquids considered for this purpose, carbon dioxide is recognized as the most commonly used solvent in SFE processes, mainly because its relatively mild critical conditions (31.3 °C and 73.8 bar). In addition, CO_2_ has other desirable properties as it is non-flammable, non-explosive, available in high purity, relatively cheap, non-toxic (*generally recognized as safe*-GRAS) and inert [49], which makes it very suitable for the SFE processes.

Extraction and separation are the two main steps of the SFE process. The solvent is first compressed to the working pressure and temperature, which allows its diffusion into the plant matrix and mass transfer from the solid to the liquid phase [50]. The separation of supercritical fluid and lipid extracts can be easily achieved by lowering the pressure and/or temperature in the separator, releasing the gaseous fluid and recovering the solvent-free extracts. Some SFE plants are equipped with two or more separators when it is necessary to separate the extracts into different fractions immediately by choosing the appropriate combination of pressure and temperature [51]. Moreover, the recirculation of the solvent in industrial scale SFE processes could be achieved to improve their efficiency.

### 4.2. Influence of SFE Parameters

Efficiency, yield and selectivity of the SFE process depend largely on the choice of the main process parameters (pressure, temperature, solvent flow rate, particle size, etc.). Therefore, the application of SFE requires the use of a very specific high pressure process equipment with integrated control of the factors affecting the process. SFE parameters must be selected according to the physicochemical properties of the target compounds and the process should be frequently optimized for each case study to achieve maximum yield of the target compound(s). Solvent solubility is directly affected by pressure and temperature; therefore, they are often considered the most important SFE parameters. Increasing the solvent density and thus improving the solubility is associated with increasing the pressure. This leads directly to a higher total extraction yield, which in certain cases can represent a significant improvement in process efficiency. However, the increased solubility leads to the co-extraction of other lipophilic compounds. For example, an SFE extract of coriander obtained at 100 bar and 40°C would be rich in volatile terpenoids (mainly linalool), whereas SFE at 300 bar and 40°C would tremendously improve the total yield due to co-extraction of fatty oil, resulting in low selectivity towards terpenoids [52]. This leads to dilution of active compounds with concomitants and requires further complications in purification of the extract. Although increasing the process pressure could decrease the selectivity towards the target compounds, in certain cases the co-extracted compounds could have a positive effect on the bioactivity of the extracts obtained [24]. 

Contrarily to pressure, an increase in temperature has a negatively effect on solvent density, often resulting in poor extraction yields. However, temperature affects both solvent properties and vapor pressure of solutes, which could improve their solubility and lead to higher yield [53]. Since the effects of temperature on solvent density and vapor pressure of solutes are contradictory, the final effect on total extraction yield cannot be easily predicted and must be observed experimentally. This suggests that a suitable selection of solvent selectivity could be made by adjusting the pressure and temperature. According to the literature data, most SFE experiments are carried out at a pressure of 100–400 bar and a temperature of 40–60 °C range [21], which allows a density of 200 to 900 kg m^−3^ of supercritical carbon dioxide, making it a suitable solvent for the isolation of lipid compounds. Understanding the thermodynamic and kinetic nature of the SFE process is critical to ensure high selectivity of extraction and reduce the possibility of co-extraction of non-target compounds [22]. 

For example, EO terpenoid-rich extracts may often be contaminated with other non-volatile lipids that could affect the bioactivity of the extract and degrade its quality. It should be emphasized that the selectivity of supercritical carbon dioxide is limited to different classes of lipid compounds and is very limited for the recovery of polar and moderately polar bioactives. The selectivity and solubility of supercritical carbon dioxide can be modified by adding various co-solvents, which can be either polar (ethanol and methanol) or non-polar (hexane and methylene chloride). Since non-polar organic solvents have low selectivity, their use as co-solvents could have a positive effect on the total extraction yield. However, their use is not often recommended because it affects the green character of the SFE. On the other hand, ethanol is most commonly used as a co-solvent for SFE due to its low miscibility with carbon dioxide, lower toxicity and easy removal from the extract [22]. This approach directly improves the spectrum of target compounds to moderately polar bioactives that can be successfully isolated using SFE. One might assume that SFE has very limited applicability for the recovery of polar plant bioactives such as anthocyanins. However, there are numerous applications of supercritical CO_2_ with the addition of ethanol as a co-solvent for the preparation of anthocyanin-rich extracts, which have been discussed elsewhere [54].

In addition to pressure and temperature, solvent flow rate and extraction time are also studied as important parameters of SFE. Mass transfer parameters such as axial dispersion, convective mass transfer coefficient and accumulation of extracted compounds in the supercritical phase are highly influenced by the flow rate of solvent [16]. Increasing the flow rate of the solvent directly improves mass transfer by increasing the concentration gradient created by the constant supply of fresh solvent. However, in some cases, this factor could lead to a decrease in the total extraction yield. Excessive solvent flow may reduce the contact time between solvent and matrix and prevent mass transfer caused by internal diffusion. Therefore, solvent flow and its consumption must be evaluated from both technological and economic points of view, as increased solvent flow could cause unnecessarily high solvent consumption.

Time is also an essential attribute of any technological process. Increasing the extraction time would usually lead to an asymptotic increase in the total extraction yield, and time is closely related to solvent consumption. Therefore, optimization of SFE can be performed by selecting the total extraction yield or the yield of target compounds as the responses [24]. Most studies on the optimization of SFE are based on this approach, which is efficient for laboratory scale studies but has severe limitations in industrial processes. The process optimized by this approach could not often be scaled up to industrial scale because it is not economically feasible to perform extractions up to a diffusion-controlled time period due to the excessive time required. Therefore, recent studies have proposed a combined approach in which the extraction kinetics are modeled in a first step and then the initial gradient is optimized either by artificial neural networks (ANN) or by response surface methodology (RSM) [23,55,56,57,58]. In addition to the above process parameters, the SFE process is highly dependent on the properties of the plant matrix, particle size and distribution and post-harvest treatment of the material, which must be considered and evaluated for each case study.

### 4.3. Application on Recovery of Bioactive Compounds

The application of SFE for the recovery of lipophilic bioactives is associated with severe drawbacks, as the processes must be performed at high pressure to maintain the solvent in a supercritical state. Compared with conventional SLE methods, this directly requires higher operating costs [59]. SFE requires rather complex process equipment compared with conventional techniques, resulting in high capital costs, especially for industrial scale processing equipment. Prado et al. [60] calculated the techno-economic aspects of SFE of grape seed oil. According to their study, the investment costs for SFE equipment are about USD 100,000, USD 300,000 and USD 1,150,000 for a processing plant with two extractors of 5, 50 and 500 L, respectively. Therefore, a limiting factor for further use of SFE processes is the price of the final product [61]. However, a process that benefits workers, industrial companies, the economy and consequently the general population and the environment should be the main objective and rational reasons for the increasing use of this promising technique [62]. SFE has already found numerous industrial applications on a large scale, for example, in the food processing (decaffeination of coffee and tea), production of food ingredients (hops and aromas, colourants, vitamin-rich extracts, etc.), nutraceuticals, pharmaceuticals from natural products and removal of pesticides from plant material [61,63]. It should be emphasized that life cycle assessments and techno-economic calculations should be carried out when developing SFE processes, especially on an industrial scale [64].

Data from a thorough literature review of scientific reports dealing with the use of lipophilic extracts in meat products relieved that SFE was frequently used to isolate these extracts. Hence, it is reasonable to assume that SFE extracts from other fruits, aromatic and medicinal plants, which are also rich in compounds with some antioxidant or antimicrobial activity, could also be used to somewhat extend the shelf life of meat products. Lipophilic extracts with their main constituents that have already been used in meat processing are listed in Table 1. Plant extracts that could potentially be used to extend the shelf life of meat products based on their chemical composition are also listed in Table 1. The chemical composition of the lipophilic extracts obtained is largely determined by factors related to plant origin (e.g., plant species, plant organ, environmental conditions, plant nutrition) and processing technology (e.g., harvest date, plant drying, milling, SFE process parameters, storage conditions). Depending on the plant species, different plant parts are subjected to SFE to obtain lipophilic extracts. For example, leaves of *Mentha piperita* L. (mentha), *Rosmarinus officinalis* L. (rosemary), *Lippia thymoides* L. (lippia), and *Origanum vulgare* L. (oregano), leaves and flowering tops from *Ocimum basilicum* L. (basil), aerial parts of *Satureja montana* L. (winter savory), seeds of *Coriandrum sativum* L. (coriander) and *Piper nigrum* L. (pepper), rhizomes from *Zingiber officinale* L. (ginger), flowers of *Lavandula angustifolia* L. (English lavender), peels of *Citrus sinensis* L. (orange) and *Citrus tangerine* L. (tangerine) or fruits of *Carum copticum* L. (ajwain) and *Malpighia Punicifolia* L. (acerola) (Table 1).

The raw material for the extraction of lipophilic compounds may be plants grown specifically for essential oil production, or by-products of the tea or fruit processing industries. For example, orange and tangerine peels left over after juice processing could be used to produce extracts enriched in terpenoids and fatty acids [76,77]. Additionally, powdered *Salvia officinalis* L. (sage) and *Thymus serpyllum* L. (wild thyme), which is not suitable for the production of filter tea bags due to the low-particle size of these fractions, is discarded as a by-product [25,36]. However, herbal dust can be used as a raw material for SFE to isolate valuable lipophilic compounds such as terpenoids. 

The applied SFE parameters for the recovery of lipophilic compounds can vary to a great extent. For example, CO_2_ pressure, which is one of the most important SFE parameters, was set at 71 bar to recover 2-furancarboxaldehyde,5-(hydroxymethyl) and other compounds from acerola [67], whereas 350 bar was used for the extraction of α-terpineol, carvacrol, borneol, thymol and other lipophilic compounds from wild thyme [36]. Temperature, another important parameter of SFE, varied to a lesser extent and all SFEs were carried out in the range of 35 °C to 60 °C (Table 1). The lowest temperature was used for the recovery of limonene from orange peel, since the solubility of limonene was highest when the processing parameters were set at 35 °C and 125 bar [76]. The highest applied temperature (60 °C) was chosen in the SFE for the recovery of lipophilic compounds from oregano and ginger [65,66]. 

The time taken to extract the plant material may also vary. A total of 40 min (dynamic stage) was required to isolate an extract containing 25.38% β-caryophyllene, 15.64% limonene and 13.63% sabinene in volatile phase from pepper [75]. On the other hand, 240 min were applied to obtain an extract containing 313.57 mg/g carvacrol from winter savory [66], 155 mg/g linalool in an extract from coriander [68], a sample containing 379.00 mg/g linalool from basil [73], or a sage extract containing 20.14% and 18.24% epirosmanol and viridiflorol in the volatile phase, respectively [25]. Total extraction yield is highly dependent on the applied process parameters used and the plant material. In certain cases, the extraction yield could be very low, as in tangerine (1.34 g/100 g) [77] and lippia (1.63 g/100 g) [74], intermediate (9.82 g/100 g) when SFE was used to obtain lipophilic compounds from lavender flowers [72], or relatively high (38 g/100 g) when oregano leaves were isolated [65] (Table 1). 

Similar to the total extraction yield, the chemical composition of the lipophilic extracts correlates strongly with the plant material, but also with the SFE parameters. Carvacrol and/or thymol, which are cyclic monoterpenoids and derivatives of p-cymene, can be found in significant amounts in oregano leaves [65], ajwain fruits [70], aerial parts of winter savory [66], lippia leaves 74] and thyme [36]. Monoterpenoids such as menthol, menthole and their respective stereoisomers (e.g., isomenthole) can be isolated in larger amounts from mentha leaves [69]. Lavender flowers, coriander seeds and basil leaves, and flowering tops can be utilized as raw material to obtain a lipophilic extract enriched in linalool (noncyclic monoterpenoid) [68,73], whereas lavender flower extract may also contain large amounts of linalool acetate ester (linalyl acetate) [72]. Monoterpene ketones such as camphor or α-thujone can be extracted from sage [25], whereas camphor has also been isolated in significant amounts from rosemary leaves [71]. 

Mattje et al. [66] reported that α-zingiberene, β-sesquiphellandrene, α-farnesene and β-bisabolene can be successfully extracted from ginger rhizomes. Orange peels could be used as a good source to obtain an extract composed almost entirely of limonene (99.5%) when the SFE parameters (t = 35 °C and 125 bar) were appropriately chosen (Table 1). Terpenoids are not the only lipophilic compounds that could be isolated by SFE. Xiong and Chen [77] reported that the chemical composition of the extract obtained from tangerine peels consisted mainly of linoleic acid (32.30%), oleic acid and n-hexadecanoic acid (14.62%), whereas terpenoids such as α-farnesene (3.32%) were detected in lower amounts (Table 1). Other notable compounds include β-caryophyllene (bicyclic sesquiterpene) found in lipophilic extracts of rosemary leaves, pepper seeds and mentha leaves [69,71], α-terpineol (cyclic monoterpenoid alcohol) isolated from thyme [36]. Additionally, various furans were obtained from acerola [67], limonene (cyclic monoterpene) and sabinene (bicyclic monoterpene) from pepper [75], and epirosmanol (diterpene lactone) and viridiflorol (sesquiterpenoid) from sage [25].

## 5. Natural Extracts Obtained by SFE and Their Application in Meat Products

The main studies conducted on the application of natural extracts obtained by SFE in meat and meat products are summarized in Table 2 and Table 3. The extracts were isolated from medicinal and aromatic plants (e.g., Echinacea spp., oregano, rosemary, sage, ginger, winter savory, wild thyme) and from fruit berries (e.g., tamarillo, acerola, pomegranate, chokeberry).

*Echinacea* spp. is a well-known medicinal plant from North America with strong antioxidant potential [14]. Gallo et al. [14] investigated the use of Echinacea angustifolia extracts as a potential natural antioxidant in chicken burgers. The plant extracts were obtained by conventional (water/ethanol) and emerging (SFE) extraction techniques. Both types of plant extracts were added to chicken meat at a concentration of 2 mL/kg in. The obtained meat product was cooked and stored at −20 °C for 10 days. In terms of strong antioxidant potential, both kinds of extracts were effective in reducing lipid and protein oxidation in chicken burger during storage, but the extract obtained by SFE showed higher efficiency and better selectivity than the conventional one. Additionally, the aroma and texture of the chicken burgers with *Echinacea* extracts were acceptable to consumers. 

Oregano (*Origanum vulgare* L.) is used in food industry as a flavouring agent and natural additive because of its flavouring properties, which are due to its essential oil. Oregano essential oil is mainly rich in terpenoid compounds (e.g., carvacrol, thymol, *p*-cymene and γ-terpinene), which have obvious preservative potential in various foods [65].

Rosemary (*Rosemarinus officinalis* L.) is known as a medicinal and aromatic plant with high content of bioactive compounds such as rosmarinic acid, carnosic acid and carnosol, which have significant antioxidant, antiviral, antibacterial and anti-inflammatory potential [65]. 

SFEs extracted from oregano and rosemary were added to fish patties (Atlantic salmon) to reduce cholesterol oxidation. The results of this study indicate that the addition of oregano and rosemary extracts (1 and 3 g/kg) effectively reduced cholesterol oxidation products (7α-hydroxycholesterol, 7β-hydroxycholesterol and 7-ketocholesterol) in fish patties during 14 days of cold storage [65]. 

Sage (*Salvia officinalis* L.) has long been used as a medicinal and aromatic plant, for its specific flavour and its strong antioxidant and antimicrobial effects. The predominant compounds in sage essential oil are monoterpene ketones, although its strong antioxidant potential is mainly associated with the presence of diterpene polyphenols [24,25]. Šojić et al. [25] evaluated the effect of extracts (Soxhlet and SFE) isolated from sage herbal dust on the quality and safety of fresh pork sausages during 8 days of cold storage. It was found that the extracts obtained by Soxhlet extraction and SFE added at 0.050, 0.075 and 0.100 µL/g decreased lipid oxidation and total plate count in fresh pork sausages. Moreover, the extract obtained with SFE was more effective against microbial growth and provided better sensory quality of fresh pork sausages, indicating the advantages of novel extraction technique. It should also be mentioned that the sage herbal dust extract obtained by SFE effectively reduced the growth of some pathogenic bacteria (*Listeria monocytogenes* and *Escherichia coli*) in ground pork [16,26]. Danilović et al. [26] observed that sage extract obtained by SFE at a concentration of 0.4, 0.6 and 1 μL/g can reduce the growth of *E. coli* in ground meat during 8 days of cold storage for. In another study, Danilović et al. [16] showed that the same extract at a concentration of 0.300 µL/g, can effectively reduce the number of *L. monocytogenes* and, consequently extend the shelf-life of ground meat by up to 6 days.

Ginger (*Zingiber officinale*) has a high content of bioactive compounds, including gingerol and its derivatives, with a strong antioxidant potential [66]. The essential oil and extracts obtained from ginger have great potential as an alternative to synthetic antioxidants in foods. Mattje et al. [6] examined the effect of application of ginger extracts (0.2%) on the quality of fish burgers (Oreochromis niloticus). The fish burgers were cold stored for 8 days. The extracts were obtained by conventional hydrodistillation and by the emerging SFE technique. Both extracts reduced lipid oxidation in the beef burgers. It is also worth highlighting that the extract obtained by SFE showed better antioxidant potential than the conventional one, probably due to as the higher content of gingerol compounds. In terms of sensory properties, Mattje et al. (2019) found that the use of ginger extract at 0.2% had a negative impact on the aroma of the burger, especially in the batches prepared with the extract obtained by SFE. The use of lower ginger extract content is important to maintain the overall quality of the meat products.

Winter savory (*Satureja montana* L.) is known as a medicinal plant with diverse pharmacological activities due to its chemical form. The most abundant compounds in the essential oil of winter savory are phenolic terpenoids, thymol, and carvacrol, which have potent antioxidant and antimicrobial potential [37]. Šojić et al. [37] studied the effect of winter savory essential oil and extract (0.075 and 0.150 µL/g) on lipid oxidation and microbial growth of fresh pork sausages stored at 3 ± 1 °C for 8 days. The essential oil was obtained by hydrodistillation, whereas the extract was obtained by SFE. In the above-mentioned study, Šojić et al. [37] found that the application of both types of extracts delayed lipid oxidation and microbial growth and prolonged the shelf-life of fresh pork sausages. It should be emphasized that the extract obtained by SFE had a stronger antioxidant and antimicrobial potential than the essential oil, most probably due to the higher concentration of co-extracted non-volatile lipids. It was found that terpenoid-rich extracts of winter savory could be used as a natural food additive in cured meat products. In another study, Jokanović et al. [15] investigated the effect of winter savory extracts on the shelf-life of marinated meat. Winter savory extract (0.200 µL/g) obtained by SFE showed a strong protective effect against lipid and protein oxidation and preserved the sensory quality of precooked pork chops during 6 days of cold storage for [15].

Extracts of wild thyme (*Thymus serpyllum* L.) are characterized by the presence of various types of bioactive compounds (e.g., terpenoids, tannins, flavonoids) that have significant antioxidant, antitumor, antimicrobial, and hypoglycemic potential [36]. Šojić et al. [36] investigated the application of wild thyme by-products (0.075 and 0.150 μL/g) obtained by SFE on the physico-chemical (colour, lipid and protein oxidation), microbial and sensory quality of ground pork patties during cold storage for 3 days. The results suggested that wild thyme by-products obtained by SFE can be used as natural additive in fresh meat products. They efficiently reduced discolouration, lipid and protein oxidation, and microbial growth (mesophilic bacteria and *Enterobacteriaceae*). The strong preservative effect of these extracts could be related to their chemical form: high content of carvacrol and thymol.

Berries are predominantly rich in phenolic compounds with strong antioxidant potential, and therefore the use of various fruit extracts in meat products suggests good antimicrobial potential [39]. Tamarillo (*Solanum betaceum* Sendtn) is an indigenous fruit from South America with significant content of bioactive compounds, including carotenoids, anthocyanins and other phenolic compounds with important preventive, biological and therapeutic properties [39]. Castro-Vargas et al. [39] studied the use of tamarillo extracts in cooked beef. Two types of extracts were prepared: an extract obtained by conventional Soxhlet extraction, and an extract obtained by SFE. The extracts were used in the processing of cooked beef to reduce lipid oxidation during 9 days of cold storage for. Castro-Vargas et al. [39] found that the application of tamarillo extract obtained by SFE effectively suppressed lipid oxidation in cooked beef. The highest antioxidant potential was found for extract obtained with supercritical CO_2_ and the addition of EtOH as a co-solvent. The use of EtOH as a co-solvent resulted in an increase in the content of polar phenolic compounds with strong antioxidant potential. 

The extract of acerola (*Malpighia punicifolia*), long used in folk medicine, has been studied in vitro for its antibacterial activity. Tremonte et al. [67] investigated the antimicrobial potential of acerola fruit extracts obtained by SFE in water buffalo meat during 21 days of cold storage. Acerola was added to the meat at 0.0063, 0.0125, 0.025 and 0.05%. The results of this study suggested that acerola fruit extract suppressed the growth of *Brochothrix thermosphacta* and *Pseudomonas* spp. in water buffalo steaks at concentrations of 0.0063 and 0.0125% and significantly reduced the initial cell count at the concentrations of 0.025 and 0.05% [67]. The strong antimicrobial potential of this extract might be related to the relatively high content of phenolic compounds. 

Kryževicūtė et al. [17] observed that raspberry pomace contains higher content of phenolic compounds than the whole fruit and pulp. Hence, raspberry pomace could be a significant reservoir of valuable phenolic compounds that have great potential as emerging antioxidant in the food industry. Kryževicūtė et al. [17] isolated extracts from raspberry pomace using SFE and pressurised liquid extraction (PLE). The extracts obtained were added to beef burgers at 0.5 and 1%. The results of this study showed that the extract obtained by PLE effectively reduced lipid oxidation in beef burgers during 26 days of cold storage. This could be the result of the high content of polyphenolic compounds, mainly ellagic acid derivatives. In contrast, the extract obtained with SFE resulted in an increase in lipid oxidation products. The prooxidant activity of this type of extract could be related to the high content of easy oxidizable PUFA. Moreover, raspberry pomace extract had no significant effect on the numbers of *B. thermosphacta*, *Pseudomonas* sp., LAB and *Enterobacteriaceae* during 26 days of storage of the beef hamburgers at 4 °C [17]. To obtain a raspberry pomace extract with strong antioxidant and antimicrobial potential in meat products, further optimization of SFE is essential.

Pomegranate (*Punica granatum* L.) peel is a major by-product of fruit processing with numerous health benefits: antioxidant, anticarcinogenic, antibacterial, anti-inflammatory, and antiatherosclerotic activities. The bioactive potential of pomegranate peels might be related to the high content of polyphenols, especially ellagitannins [19]. Silva et al. [19] investigated the effect of pomegranate peel extracts (100 ppm) obtained by SFE on the oxidative stability and colour of bluefish patties stored at 4 °C for 9 days. In order to obtained extracts enriched in bioactive compounds, sequential fractionation with supercritical-CO_2_ was performed. Two fractions of pomegranate peels were prepared: a fraction with high phenolic content and a lipid fraction. The results of this study showed that the phenol and lipid rich fractions from pomegranate peels effectively reduced lipid and protein oxidation without negatively affecting the colour changes of the fish pies. It is also worth highlighting that both fractions have higher antioxidant potential than the synthetic antioxidant butylated hydroxytoluene. The obtained results suggest that pomegranate peel fractions could be used as an effective substitute for synthetic antioxidants in fish meat. 

Additionally, chokeberries and their pomace are characterized by significant antioxidant potential due to their high content of bioactive compounds, especially polyphenols [18]. Tamkutė et al. [18] investigated the quality and safety of meat products (pork burgers and cooked ham) prepared with chokeberry pomace extract (2%) as a natural antioxidant. It was also found that the use of this extract at 2% had no negative effect on the sensory quality of the pork burgers and cooked ham. The extract was obtained from defatted chokeberries by SFE using pressurized ethanol. The results of the above study showed that chokeberry pomace extract effectively reduced lipid oxidation in pork burgers during cold storage for 16 days. The authors observed that chokeberry pomace extract efficiently reduced lipid oxidation in pork burgers during cold storage for 16 days. In conclusion, the authors suggested that chokeberry pomace extract could be used as an effective natural additive in pork products and as a promising ingredient to improve the health benefits of meat products [18,78].

## 6. Concluding Remarks and Further Challenges

Nowadays, there is a huge demand for natural antioxidants and antimicrobials, mainly due to the controversial toxicological reports on many synthetic compounds. First, the chemistry and bioactivity of natural antioxidants currently used as additives in meat products was presented. This was followed by the fundamentals of SFE as the most promising green and clean method to obtain lipophilic bioactives, as well as an overview of recent research on the isolation of extracts with strong antioxidant and/or antimicrobial activity and optimization of the process. Finally, we also processed new scientific data on the application of extracts from medicinal and aromatic plants (e.g., oregano, sage, rosemary) and from berry fruits (acerola, pomegranate, chokeberry) obtained by SFE in meat processing. 

The results presented in this review indicate that the extracts obtained by SFE can be successfully used as novel antioxidants and antimicrobial agents in various types of meat and meat products. In some studies, it was found that the use of SFE offered higher efficiency and better selectivity, and thus better antioxidant and antimicrobial potential of the newly formed extracts than conventional methods, including hydrodistillation and Soxhlet extraction. Further studies should evaluate the ability of the natural additives obtained by SFE as potential substitutes for sodium nitrite in cured meat products.

Although the interest of the scientific community in the study of natural extracts obtained by SFE is constantly growing, there is still room for further improvement to expand the applications in the industrial sector. Therefore, further investigations and challenges can be divided into several directions. First, research should focus on the production of high-quality extracts rich in bioactive compounds (essential oil, polyphenols, carotenoids, antioxidants, etc.) by SFE. In addition to innovating the SFE process and optimizing the extraction procedure, alternative plant resources (underutilized medicinal plants, food industry by-products, and agricultural wastes) should be used as raw materials for the isolation of bioactives. Another important aspect in the production of these extracts is the encapsulation process to find a suitable way to protect the bioactive compounds from possible degradation and to ensure high quality of the final product in terms of odor, solubility of bioactive compounds and improved efficacy (better bioavailability). Finally, it is expected that the number of studies dealing with the application of extracts obtained by SFE as natural additives in meat products will increase and will be studied in different meat products, as well as in different food matrices.

## Figures and Tables

**Table 1:** Plants and their parts subjected to supercritical fluid extraction to obtain extracts and major compounds in isolated extracts.

Plant	Plant Organ	Processing Parameters	Major Compounds and Concentration in Extract	Extraction Yield **	Ref.
*Origanum vulgare L.*	leaves	t = 150 min q = 600 kg/h I stage S = CO_2_ p = 300 bar T = 50 °C II stage S = CO_2_ p = 160 bar T = 60 °C	Carvacrol = 50.1% * Thymol = 6.9% * Thymoquinone = 5.1% *	38 g/100 g	[65]
*Satureja montana L.*	aerial parts	S = CO_2_ p = 300 bar T = 40 °C t = 240 min q = 0.2 kg/h	Carvacrol = 313.57 ± 8.66 mg/g *p*-Cymene = 131.83 ± 5.07 mg/g	4.14 g/100 g	[37]
*Zingiber officinale L.*	rhizomes	S = CO_2_ p = 250 bar T = 60 °C	α-Zingiberene = 42.1% * β-Sesquiphellandrene = 17.0% * α-Farnesene = 11.0% * β-Bisabolene = 9.8% *	/	[66]
*Thymus serpyllum L.*	/	S = CO_2_ p = 350 bar T = 50 °C t = 180 min q = 0.3 kg/h	α-Terpineol = 77.47 ± 0.76 mg/g Carvacrol = 63.25 ± 0.22 mg/g Borneol = 41.33 ± 0.52 mg/g Thymol = 19.64 ± 0.12 mg/g	2.93 g/100 g	[36]
*Salvia officinalis L.*	/	S = CO_2_ p = 100 bar T = 40 °C t = 240 min q = 0.2 kg/h	Epirosmanol = 20.14% * Viridiflorol = 18.24% * Camphor = 107.48 mg/g α-Thujone = 91.54 mg/g	/	[25]
*Malpighia Punicifolia L.*	fruit	P = 71 bar T = 42.2 °C	2-Furancarboxaldehyde, 5-(hydroxymethyl) = 43.79% *	/	[67]
*Coriandrum sativum L., Apiaceae*	seeds	S = CO_2_ p = 200 bar T = 55 °C t = 240 min q = 0.4 kg/h	Linalool = 155 mg/g Camphor = 7.2 mg/g	7.0 g/100 g	[68]
*Mentha piperita L.*	leaves	S = CO_2_ p = 100 bar T = 40 °C t = 180 min q = 0.3 kg/h	Menthol = 330.31 ± 2.22 mg/g Menthone = 66.28 ± 0.12 mg/g Isomenthole = 38.82 ± 0.22 mg/g β-Caryophyllene = 35.71 ± 0.11 mg/g Eucalyptol = 31.52 ± 0.11 mg/g	/	[69]
*Carum copticum L.*	fruits	S = CO_2_ p = 167 bar T = 40 °C t = 90 min q = 8 mL/min	Thymol= 63.75% *	2.78 ± 0.04 g/100 g	[70]
*Rosmarinus officinalis L.*	leaves	S = CO_2_ p = 172.4 bar T = 40 °C t = 90 min q = 126.24 ± 20.83 mL/min	Camphor = 28.42% * β-Caryophyllene = 12.18% * Eucalyptol = 10.60% *	2.53 g/100 g	[71]
*Lavandula angustifolia L.*	flower	S = CO_2_ p = 180 bar T = 50 °C t = 10 (static) + 60 (dynamic) min q = 2 mL/min	Linalool = 42.74% * Linalyl acetate = 23.25% *	9.82 g/100 g	[72]
*Ocimum basilicum L.*	leaves and flowering tops	S = CO_2_ p = 300 bar T = 40 °C t = 240 min q = 0.2 kg/h	Linalool = 379.00 mg/g Camphor = 17.10 mg/g α-Terpineol = 17.00 mg/g	2.07 g/100 g	[73]
*Lippia thymoides L.*	leaves	S = CO_2_ p = 300 bar T = 50 °C t = 30 (static) + 120 (dynamic) min q = 8.85 10^−5^ kg/s	Thymol = 83.64 ± 0.29% *	1.63 ± 0.01 g/100 g	[74]
*Piper nigrum* *L.*	seeds	S = CO_2_ p = 300 bar T = 40 °C t = 30 (static) + 40 (dynamic) min q = 2 mL/min	β-Caryophyllene = 25.38 ± 0.62% * Limonene = 15.64 ± 0.15% * Sabinene = 13.63 ± 0.21% *	/	[75]
*Citrus sinensis L.*	peels	S = CO_2_ p = 125 bar T = 35 °C	Limonene = 99.5% *	/	[76]
*Citrus tangerine L.*	peels	S = CO_2_ p = 140 bar T = 40 °C t = 147 min q = 20 L/h	Linoleic acid = 32.30% * Oleic Acid = 20.42% * n-Hexadecanoic acid = 14.62% * α-Farnesene = 3.32% *	1.34 g/100 g	[77]

S—solvent; p–pressure; T—temperature; t—extraction time; q—solvent flow; *—concentration in volatile fraction; **—maximal extraction yield.

**Table 2:** Antioxidant effects of extracts obtained by SFE in meat and meat products.

Plant Extract	Dose	Meat/Meat Product	Storage	Effect	Reference
*Echinacea* *angustifolia*	2 mL/kg	Frozen chicken meat	−20 °C, 10 days	Reduced lipid and protein oxidation	[14]
Tamarillo epicarp	200 mg/kg	Cooked beef	4 °C, 9 days	Reduced lipid oxidation	[39]
Oregano	1 and 3 g/kg	Fish patties	4 °C, 9 days	Reduced cholesterol oxidation	[65]
Raspberry pomace	0.5 and 1%	Beef burger	4 °C, 26 days	Increased lipid oxidation; no effect in preserving colour	[17]
Sage herbal dust	0.05, 0.075 and 0.100 µL/g	Fresh pork sausages	3 °C, 8 days	Reduced lipid oxidation; preserved sensory characteristics	[25]
Ginger	0.2%	Fish burger	4 °C, 8 days	Reduced lipid oxidation; Negative effect on sensory characteristics	[66]
Winter savory	0.075 and 0.150 µL/g	Fresh pork sausages	3 °C, 8 days	Reduced lipid and protein oxidation; preserved sensory characteristics	[37]
	0.2 µL/g	Precooked pork chops	4 °C, 6 days	Reduced lipid and protein oxidation; preserved colour, texture and sensory characteristics	[15]
Wild thyme by-product	0.075 and 0.150 µL/g	Ground pork patties	4 °C, 6 days	Reduced lipid and protein oxidation; preserved colour	[36]
Pomegranate peel	100 ppm	Bluefish patties	4 °C, 9 days	Reduced lipid and protein oxidation; no effect in preserving colour	[74]
Chokeberry extract	2%	Raw pork burgers and cooked ham	4 °C, 7 days (burger) and 13 days (ham)	Reduced lipid oxidation (burger); no negative effect on sensory characteristics (burger and ham)	[18]

**Table 3:** Antimicrobial effect of supercritical fluid extracts in meat and meat products.

Plant Extract	Dose	Meat/Meat Product	Storage	Effect	Ref.
Acerola	0.0063, 0.0125, 0.025 and 0.05% (*w/v*)	Water buffalo steaks	4 °C, 21 days	*B. thermosphacta* and *Pseudomonas* spp.	[66]
Raspberry pomace	0.5 and 1%	Beef burger	4 °C, 26 days	No significant effect on the number of *B. thermosphacta*, *Pseudomonas* sp., LAB ^1^ and *Enterobacteriaceae*	[17]
Sage herbal dust	0.05, 0.075 and 0.1 μL/g	Fresh pork sausages	3 °C, 8 days	Reduction in AMB ^2^ count	[25]
Winter savory	0.075 and 0.150 µL/g	Fresh pork sausages	3 °C, 8 days	Reduction in AMB count and Enterobacteriacea	[37]
Wild thyme by-product	0.075 and 0.150 µL/g	Ground pork patties	4 °C, 6 days	Reduction in total plate count, *Enterobacteriaceae* and LAB	[36]
Chokeberry pomace extract	2%	Pork slurry	4 °C, 16 days	bacteria (LAB), and aerobic mesophilic bacteria (AMB) Reduction in the growth of *L. monocytogenes*, *B. thermosphacta*, *P. putida*, and AMB	[77]

^1^ LAB—lactic acid bacteria; ^2^ AMB—aerobic mesophilic bacteria.
